# Invasive micropapillary carcinomas arising 42 years after augmentation mammoplasty: A case report and literature review

**DOI:** 10.1186/1477-7819-6-33

**Published:** 2008-03-14

**Authors:** Yuko Tanaka, Isamu Morishima, Kazunori Kikuchi

**Affiliations:** 1 Department of Breast and Thyroid Surgery Tsukuba Medical Center Hospital. 1-3-1, Amakubo, Tsukuba-city, Ibaraki, 305-0005, Japan; 2 Department of *Pathology, Tsukuba Medical Center Hospital. 1-3-1, Amakubo, Tsukuba-city, Ibaraki, 305-0005, Japan

## Abstract

**Background:**

There has been no definitive consensus regarding the causal relationships between foreign bodies in the breast and carcinogenesis. This report describes the first case of invasive micropapillary carcinomas after augmentation mammoplasty. Multiple tumors located in immediate contact with the siliconomas suggested a causal link between the siliconomas and carcinomas.

**Case presentation:**

This report presents the case of a 64-year-old female who underwent liquid silicone injections for augmentation mammoplasty 42 years previously. Eight years before admission, siliconomas of the left breast were removed due to pain and discomfort. The patient visited the hospital for further treatment of newly diagnosed carcinoma of the left breast. Images showed multiple tumors located in various areas of the left breast. The pathological findings of the left breast showed each tumor to be solitary and not continuous with the others. The tumors were diagnosed to be invasive micropapillary carcinomas, and they all came into immediate contact with the residual siliconomas. The siliconomas were therefore suspected to have played a causative role in the development of the breast cancer.

**Conclusion:**

This rare case of multiple invasive micropapillary carcinomas following augmentation mammoplasty provides evidence that siliconomas may lead to carcinomas. Although a causal relationship was not established unequivocally, we review evidence that suggest silicone gel may cause cell damage responsible for carcinoma development.

## Background

Although breast cancer after augmentation mammoplasty has been reported and the causal relationships between foreign bodies in the breast and carcinogenesis have been reviewed, so far no definitive consensus opinion has been obtained [1-11]. This report describes a unique case of multiple invasive micropapillary carcinomas (IMPCs) of the breast arising 42 years after augmentation mammoplasty by the injection of liquid silicone. No cases of IMPC after augmentation mammoplasty have ever been reported. In this case, the multiple tumors were located in immediate contact with the siliconomas, thus suggesting a link between the siliconomas and the carcinomas.

## Case presentation

A 64-year-old woman underwent liquid silicone injections for augmentation mammoplasty 42 years previously. Eight years prior to admission, siliconomas were removed due to discomfort. She visited a hospital with the chief complaint of a painful mass in her left breast. The mass was resected and a histopathological examination revealed the tumor to be an invasive micropapillary carcinoma. The surgical margin was positive for malignant cells and she visited the hospital for further treatment. She was a healthy-looking woman. The left breast was craggy and it came in contact with the axilla, which thus made it difficult to palpate the tumors. No breast tumor was palpable on the other side. The laboratory parameters did not show any abnormalities and there was no evidence of distant metastasis. She was not on any medication. She had never taken oral contraceptives nor received hormonal therapy. She had experienced three pregnancies and delivered once. Her family history revealed no malignancies.

A subsequent dynamic magnetic resonance imaging (MRI) examination with Gadolinium (Gd)-DTPA enhancement demonstrated the four tumor shadows with similar enhancement at distant portions. Because it was unlikely that four malignant tumors existed at the same instant, they were thus considered to be coexistent malignant tumors and siliconomas. Ultrasonography revealed masses with an irregular shape and contour, extensive hypoechogenicity or shadowing. The tumors with a heterogeneous internal echo with a slight degree of Doppler signaling were considered to be malignant tumors; those with homogeneous internal hypoechogenicity with no Doppler signaling were considered to be siliconomas.

**Figure 1:**
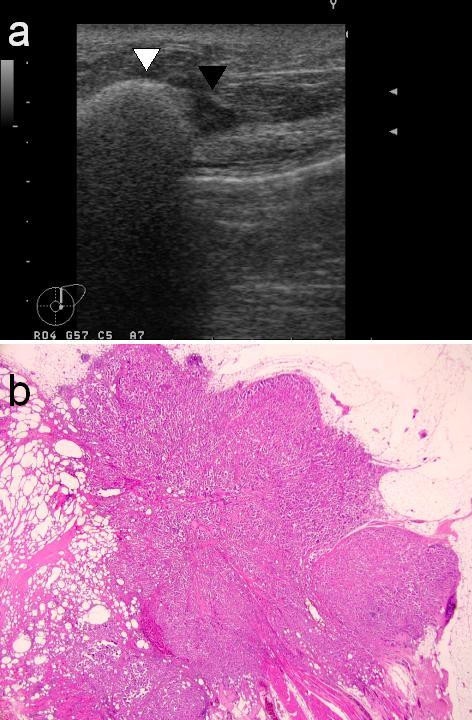
a) Ultrasonography of the left breast showing the siliconoma (white triangle) and the tumor (black triangle). b) Microscopic appearance of the siliconoma and the tumor correlated with the ultrasonographic image (hematoxylin-eosin stain, low-power field).

**Figure 2:**
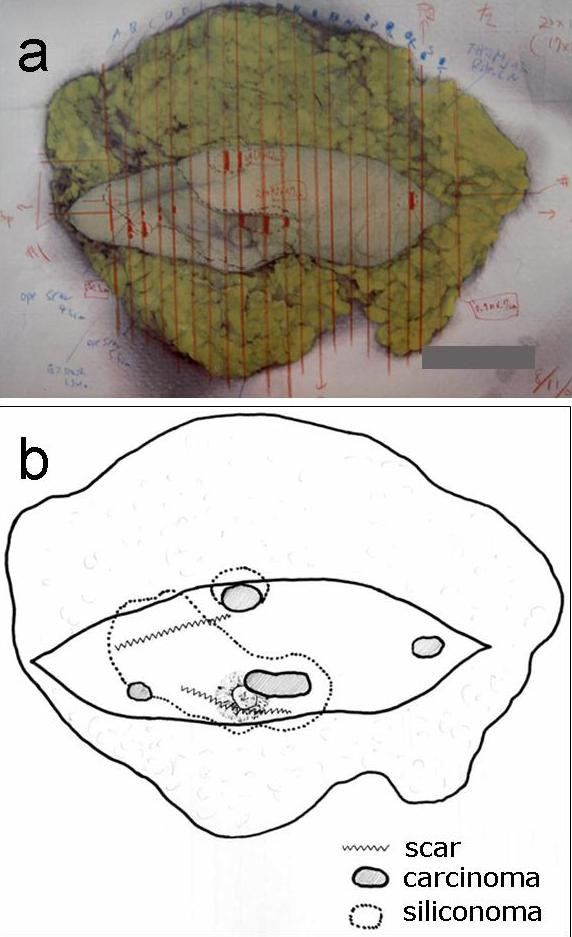
**a) Macroscopic appearance of the left breast specimen. b) Schematic drawing of the breast specimen. Siliconomas were spread extensively within the breast.** Three tumors were identified to come in contact with the siliconomas.

**Figure 3:**
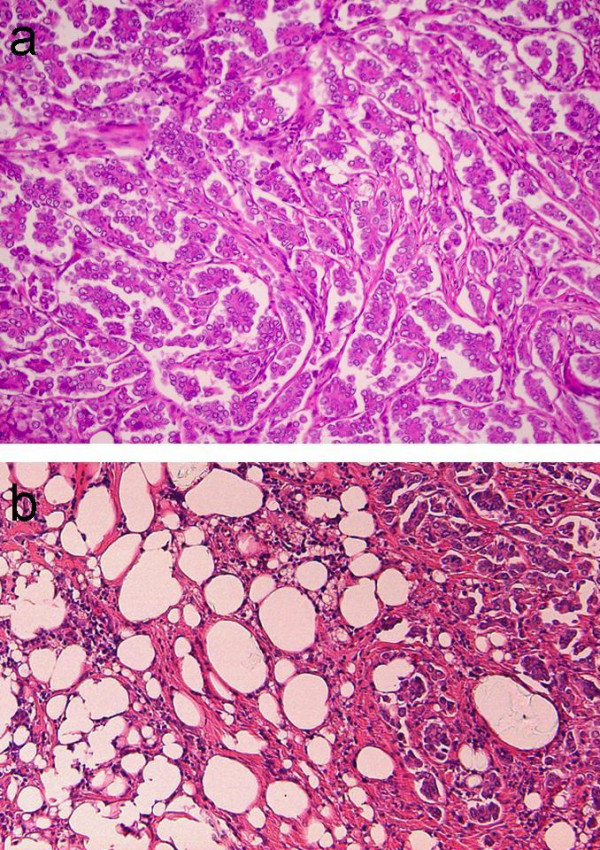
a) Microscopic appearance of the tumor diagnosed as IMPC (hematoxylin-eosin stain, high-power field), showing neoplastic cell clusters floating within clear spaces defined by a network of loose fibrocollagenous stroma. b) Microscopic appearance of the siliconoma on the border of the tumor (hematoxylin-eosin stain, high-power field), showing collections of rounded vacuoles with lipid droplets along with macrophages and foreign-body giant cells.

A left-sided mastectomy and complete axillary lymph node dissection was thus performed. The histopathological findings of the mastectomy specimen were as follows. The siliconomas were observed to be spread around the operational scar. Three tumors were identified, all in immediate contact with the siliconomas as indicated by ultrasonography (Figure 1a, b), which measured 12 mm on the upper side of the breast, 3 mm on the lateral side and 20 mm on the subareolar area. A tumor measuring 9 mm in diameter was located on the medial side, but had no connection with the siliconomas (Figure 2). In each tumor, neoplastic cell clusters floating within clear spaces defined by a network of loose fibrocollagenous stroma were recognized (Figure 3a), and the tumors were diagnosed as IMPCs. Scirrhous carcinoma components were also seen in each tumor. The malignant cells of the three tumors had contact with collections of rounded vacuoles of varying sizes (Figure 3b). Lipid droplets were contained in these vacuoles along with macrophages and foreign-body giant cells. In addition, lymphatic invasion was observed in all tumors and perineural invasion was seen for the medial tumor. The tumor in the subareolar area reached the fat tissues outside of the gland, the dermis and the larger muscle. Eleven of sixteen axillary lymph nodes showed tumor involvement. The histological grade, based on a modified Bloom Richardson scoring system, was intermediate. The scores for each parameter (tumor tubule formation, number of mitoses and nuclear pleomorphism) were 3, 1 and 2, respectively. Immunohistochemically, the tumors were estrogen receptor (ER) and progesterone receptor (PgR) positive and C-erbB-2 negative. Postoperatively, since the patient consistently refused to be treated with adjuvant systemic chemotherapy, radiotherapy was administered with 50Gy to the chest wall. Subsequently, endocrine therapy was administered using antiestrogens. Three years after the operation, no metastasis was recognized in any organ.

## Discussion

An invasive micropapillary carcinoma (IMPC) was initially described by Siriaunkgul in 1993, but such a case is not frequently observed. IMPC is known for its poor clinical outcome, with massive lymph node metastasis and extensive lymphatic invasion [12-14]. There is no clear explanation for the morphogenesis of this tumor or for how this particular morphology affects tumor behavior [15,16]. It is thought to be very rare for multiple foci to occur together in this subtype of tumor. In this case, three tumors displayed foci grouping. IMPCs were identified in approximately 40% of the microscopic field, thus revealing a mainly scirrhous pattern. Multifocality or multicentricity in breast cancer is defined as the presence of two or more tumor foci within a single quadrant of the breast, or within different quadrants of the same breast, respectively [17]. Determining whether the tumors were multifocal or multicentric was problematical. Multifocal or multicentric breast cancer occurring after augmentation has not been reported. Since the tumors were located in another quadrant without ductal continuity, it is therefore highly unlikely that the metastases occurred through the duct. It is conceivable that the three tumors metastasized through the lymphatic system in the breast, although a tumor in the medial side was assumed to have metastasized through a perineural route. Considering that three tumors existed along with the siliconomas, it is very likely that one of the causes of the development of carcinoma was the siliconomas or silicone. A report showed that the concentration of silicone appeared to be much higher within the tumor than in the adjacent breast tissue [2]. In the present case, the most concentrated area, the subareolar, could be a primary site, and cancer cells within the lymph flow appear to have deviated from usual lymphatic drainage pathway due to siliconomas or inflammation, and then they might have spread to various other regions.

There has been considerable speculation concerning the safety of breast augmentation, particularly regarding whether the use of silicone prostheses or silicone is associated with an increased risk of carcinoma and/or autoimmune disorders and few studies indicate that those who have undergone mammoplasty are at increased risk of developing breast cancer. However, in Europe and the United States, the practice of breast augmentation by the injection of liquid silicone has been stopped since the early 1970s and augmentation using bag prostheses now make up the majority of the augmented population. Epidemiological research addressing gel injections has not yet been performed. Therefore, the proper status of women augmented by silicone injection may still not be known. In Japan, the practice of breast augmentation by injection of liquid silicone was used from 1955 to 1965, and frequent reports of its complications result in the abandonment of this procedure in favor of bag prostheses. Furthermore, augmentation for cosmetic purpose in Japan is not covered by the health insurance system: therefore, there is insufficient medical data on this patient population. However, women who underwent cosmetic augmentation by silicone injection in the past, did later show an increased incidence of breast cancer. Among 63 Japanese patients, mammoplasty had been performed by injection in 41 cases and by implants in 9 cases as far as we could ascertain. The period from augmentation mammoplasty to the diagnosis of breast cancer ranged from 4 months-50 years. In particular, the patient who had previously undergone liquid silicone injections was diagnosed to have breast cancer more than 20 years after augmentation. The dominant histopathological classification was invasive ductal carcinoma, two cases were ductal carcinoma *in situ*, one case was medullary carcinoma and one case was inflammatory carcinoma.

When breast implants first appeared on the market in 1962, it was assumed that they were biologically inert and posed no medical risk. However, many reports have indicated a high prevalence of connective tissue disorders and cancer among implant patients. In contrast, no evidence has firmly established the long-term safety of breast implants. Accordingly, the United States Food and Drug Administration restricted the use of silicone breast implants to women seeking breast reconstruction in controlled clinical trials. Likewise, the United States Congress directed the National Institutes of Health to conduct a large follow-up study to evaluate the long-term health effects of the implant. No associations were identified between breast implants and cancer, immunological diseases, neurological problems, or other systemic diseases. Furthermore, breast cancer risk was not higher for any type of implant compared to another (e.g., silicone gel implants, saline-filled implants, double lumen implants, and other implant varieties) [5].

Results from the National Institutes of Health study are counterintuitive, in that many lines of evidence indicate that liquid silicone injections may pose health risks. First, the liquid silicone used for injection has been shown to occasionally contain additives which may have sometimes been of a non-medical grade. Second, the physiological response to liquid silicone may be different from that of vulcanized silicones, such as silicone gels [1]. Furthermore, Felix *et al *reported the tumorigenicity of silicone gels in the mouse plasmacytoma system [18]. They showed the possibility that low molecular weight silicone compounds, such as siloxanes, which are present as the result of incomplete polymerization in the preparation of silicone gels, leaking from the complex silicone gel matrix into the surrounding tissue, may be mutagenic and thus postulated that this mutagenicity may be a critical determinant of the plasmacytoma inducing potency of silicone gels. Studies on the stability of silicone gels *in vivo *have suggested that the polymeric structure of the gel deteriorates with age, thus resulting in the continuous release of low molecular weight siloxanes from the gel matrix. Extended exposure to liquid silicone may also have an unfavorable effect on mammary cells. The other linkage between mammoplasty and carcinogenesis could have been chronic inflammation. Chronic inflammation induced in tissues of other organs including the colon, stomach, esophagus, gallbladder, urinary bladder and lung can result in the formation of an adenocarcinoma [19-21]. Unfortunately, however, there is insufficient data suggesting a causal relationship between inflammation and carcinogenesis of the breast [22,23].

With respect to imaging modalities, mammography may not be a good screening tool in augmented women, because cancer in augmented women is significantly less likely to present as a mammographic abnormality in the absence of physical findings [3,10,24]. Mammography was impossible for our patient, due to small and contractural breasts. Ultrasonography with Color Doppler can be useful for distinguishing cancer from the siliconoma. In addition, MRI using dynamic Gd-DTPA enhancement was found to be useful for the diagnosis of breast cancer because of its high quality and resolution and made it possible to determine whether a lesion is malignant or not [3], although it was not possible to diagnose all four lesions as cancer.

## Conclusion

This report describes that the first case of IMPCs arising after augmentation mammoplasty. Although the causal relationship between mammoplasty and carcinogenesis was not established unequivocally, our review of previous studies uncovered considerable evidence for deleterious effects of silicone gel implantation that could be related to carcinogenesis. We thus consider it likely that the observed IMPCs were a result of mammoplasty.

## Abbreviations

Invasive micropapillary carcinoma; IMPC, magnetic resonance imaging, MRI, estrogen receptor; ER, progesterone receptor; PgR

## Competing interests

The author(s) declare that they have no competing interests.

## Authors' contributions

YT carried out literature search, drafted the manuscript. IM carried out initial assessment of the patient and helped in draft of manuscript. KK evaluated histopathological features and contributed histological part. All authors read and approved the final manuscript.

## References

[B1] Edelman DA, Grant S, van Os WA (1995). Breast cancer risk among women using silicone gel breast implants. Int J Fertil.

[B2] Maddox A, Schoenfeld A, Sinnett HD, Shousha S (1993). Breast carcinoma occurring in association with silicone augmentation. Histopathology.

[B3] Kasamaki S, Tsurumaru M, Kamano T, Kobayashi S, Hino M, Kawatsuru R (2000). A case of inflammatory breast cancer following augmentation mammoplasty with silicone gel implants. Breast cancer.

[B4] van Diese PJ, Beekman WH, Hage JJ (1998). Pathology of silicone leakage from breast implants. J Clin Pathol.

[B5] Nelson NJ (2000). Silicone breast implants not linked to breast cancer risk. J Natl Cancer Inst.

[B6] Brinton LA, Brown SL (1997). Breast implants and cancer. J Natl Cancer Inst.

[B7] Mclntosh SA, Horgan K (2007). Breast cancer following augmentation mammoplasty – a review of its impact on prognosis and management. J Plast Reconstr Aesthet Surg.

[B8] Deapen DM, Hirsch EM, Brody GS (2007). Cancer risk among Los Angeles women with cosmetic breast implants. Plast Reconstr Surg.

[B9] Bryant H, Brasher P (1995). Breast implant and breast cancer – reanalysis of a linkage study. N Engl J Med.

[B10] Skinner KA, Silberman H, Dougherty W, Gamagami P, Waisman J, Sposto R, Silverstein MJ (2001). Breast cancer after augmentation mammoplasty. Ann Surg Oncol.

[B11] Englert H, Joyner E, McGill N, Chambers P, Horner D, Hunt C, Makaroff J, O'Connor H, Russell N, March L (2001). Women's health after plastic surgery. Internal Medecine.

[B12] Siriaunkgul S, Tavassoli FA (1993). Invasive micropapillary carcinoma of the breast. Mod Pathol.

[B13] Crus CDL, Moriya T, Endoh M, Watanabe M, Takeyama J, Yang M, Oguma M, Sakamoto K, Suzuki T, Hirakawa H, Orita Y, Ohuchi N, Sasano H (2004). Invasive micropapillary carcinoma of the breast: Clinicopathological and immunohistochemical study. Pathol Int.

[B14] Nassar H, Wallis T, Andea A, Dey J, Adsay V, Visscher D (2001). Clinicopathologic Analysis of Invasive Micropapillary Differentiation in Breast Carcinoma. Mod Pathol.

[B15] Li YS, Kaneko M, Sakamoto DG, Takeshima Y, Inai K (2006). The reversed apical pattern of MUC1 expression is characteristics of invasive micropapillary carcinoma of the breast. Breast Cancer.

[B16] Nassar H, Pansare V, Zhang H, Che M, Sakr W, Ali-Fehmi R, Grignon D, Sarkar F, Cheng J, Adsay V (2004). Pathogenesis of invasive micropapillary carcinoma: role of MUC1 glycoprotein. Mod Pathol.

[B17] Coombs NJ, Boyages J (2005). Multifocal and multicentric breast cancer: does each focus matter?. J Clin Oncol.

[B18] Felix K, Lin S, Bornkamm GW, Janz S (1998). Tetravinyl-tetramethylcyclo-tetrasiloxane (tetravinyl D4) is a mutagen in Rat2λlacI fibroblasts. Carcinogenesis.

[B19] Thun MJ, Henley SJ, Gansler T (2004). Inflammation and cancer: an epidemiological perspective. Novartis Found Symp.

[B20] Il'yasova D, Colbert LH, Harris TB, Newman AB, Bauer DC, Satterfield S, Kritchevsky SB (2005). Circulating levels of inflammatory markers and cancer risk in the health aging and body composition cohort. Cancer Epidemoil Biomarkers Prev.

[B21] Weitzman SA, Gordon LI (1990). Inflammation and cancer: role of phagocyte-generated oxidants in carcinogenesis. Blood.

[B22] Zhang SM, Lin J, Cook NR, Lee IM, Manson JE, Buring JE, Ridker PM (2007). C-reactive protein and risk of breast cancer. J Natl Cancer Inst.

[B23] Lithgow D, Nyamathi A, Elashoff D, Martinez-Maza O, Convington C (2006). C-reactive protein in nipple aspirate fluid: relation to women's health factor. Nurs Res.

[B24] Miglioretti DL, Rutter CM, Geller BM, Cutter G, Barlow WE, Rosenberg R, Weaver DL, Taplin SH, Ballard-Barbash R, Carney PA, Yankaskas BC, Kerlikowske K (2004). Effect of breast augmentation on the accuracy of mammography and cancer characteristics. JAMA.

